# Network topology measures for identifying disease-gene association in breast cancer

**DOI:** 10.1186/s12859-016-1095-5

**Published:** 2016-07-25

**Authors:** Emad Ramadan, Sadiq Alinsaif, Md Rafiul Hassan

**Affiliations:** Department of Information and Computer Science, King Fahd University of Petroleum and Minerals, Dhahran, Saudi Arabia

**Keywords:** Biological networks, Machine learning, Phenotype-gene association

## Abstract

**Background:**

Massive biological datasets are generated in different locations all over the world. Analysis of these datasets is required in order to extract knowledge that might be helpful for biologists, physicians and pharmacists. Recently, analysis of biological networks has received a lot of attention, as an understanding of the network can reveal information about life at the cellular level. Biological networks can be generated that examine the interaction between proteins or the relationship amongst different genes at the expression level. Identifying information from biological networks is recognized as a significant challenge, due to the inherent complexity of the structures. Computational techniques are used to analyze such complex networks with varying success.

**Results:**

In this paper, we construct a new method for predicting phenotype-gene association in breast cancer using biological network analysis. Several network topological measures have been computed and fed as features into two classification models to investigate phenotype-gene association in breast cancer. More importantly, to overcome the problem of the skewed datasets, a synthetic minority oversampling technique (SMOTE) is adapted in order to transform an imbalanced dataset to a balanced one. We have applied our method on the gene co-expression network (GCN), protein–protein interaction network (PPI), and the integrated functional interaction network (FI), which combined the PPIs and gene co-expression, amongst others. We assess the quality of our proposed method using a slightly modified cross-validation.

**Conclusions:**

Our method can identify phenotype-gene association in breast cancer. Moreover, use of the integrated functional interaction network (FI) has the potential to reveal more information and hidden patterns than the other networks. The software and accompanying examples are freely available at http://faculty.kfupm.edu.sa/ics/eramadan/NetTop.zip.

## Background

Understanding crosstalk and feedback among oncogenic pathways is critical in order to predict and overcome resistance to targeted anticancer therapy. The topology of biological networks has increasingly been used to complement studies based on individual genes or gene sets. Several network applications are relevant to the study of pathway crosstalk in drug resistance. The identification of modules and sub–networks that are relatively isolated from the rest of the network can lead to an understanding of the direct interaction and cooperation among molecules and to more detailed or dynamic models of the network. Network topological characteristics can potentially be predictive biomarkers through network based classification [1, 2].

Protein interaction networks and gene co-expression networks potentially represent patterns of network connectivity among genes/proteins that differ between clinically relevant phenotypes. Various topological measures that identify relationships between genes, such as node degree, betweenness [3], or bridging [4], may contribute to the ability to predict phenotype-gene association.

Here, we apply several techniques for network analysis to demonstrate their utility in studying biological networks in breast cancer. We utilize network topological measures to expose the important nodes (genes/proteins) within the network, and identify marker genes (genes related to breast cancer) from gene co-expression networks, protein interaction networks, or integrated functional networks.

In the present work, we have extracted thirteen topological measurements (features) from a publicly available gene co-expression network and a protein interaction network. We then use classification models to investigate the phenotype-gene association in breast cancer. Moreover, we apply this approach to the integrated functional network of PPI and gene expression in order to investigate the hidden patterns of breast cancer that might not be revealed in the protein network or gene co-expression network.

## Related works

Gene expression datasets (not networks) have been used extensively for the purpose of phenotype-gene association, where the gene expression profiles are fed as features into the classifier [5–7].

Recently, the network–based approach has also been used for this purpose. For instance, Zhang et al. [8] proposed a network–based Cox regression model (Net-Cox). The proposed model was intended to investigate the gene expression signatures that contribute to the result of death or repetition in ovarian cancer treatment. Moreover, Ruan et al. [9] proposed a general co-expression network-based technique that allows analysis of genes and samples obtained from microarray datasets. This technique uses a rank–based network construction method, a parameter-free module discovery algorithm, and a reference network-based metric for module evaluation. The study utilized a number of different datasets for evaluation purposes, such as yeast and human cancer microarray.

Yuanfang et al. [10] proposed an approach that utilized a mouse genome-wide functional relationship network and support vector machine classifier to investigate the bone mineral density (BMD) of a phenotype related to osteoporotic fracture. Two genes were revealed (Timp2 and Abcg8) that are related to bone density defects that were not identified in other statistical methods (i.e. genome-wide association studies/quantitative trait loci).

Wu et al. [11] developed a naive Bayes classifier (NBC) to reveal a functional interaction (FI) network that combines both curated protein–protein interaction networks and pathway information.The computed FI network was used to investigate two glioblastoma multi–form (GBM) datasets and projected the cancer candidate genes onto the FI network.

## Methods

Our proposed methodology consists of four steps:

- Step 1: Extract topological measures from biological networks.
- Step 2: Identify the breast cancer signature genes.
- Step 3: Apply SMOTE in order to make a balanced dataset.
- Step 4: Use classification models in order to investigate the phenotype-gene association in breast cancer.

Details about these steps are described below:

### Topological measures

We study several topological measures in order to understand their capability in identifying disease markers from the biological network. Table 1 illustrates the relation among these measures. First, we need to define some graph (network) concepts.

**Table 1:** Topological measures

Degree–based measurements	Degree
	Coreness
	Clustering coefficient
Shortest–path–based measurements	Betweenness
	Closeness
	Proximity prestige
	Bary center score
Eigenvector–based measurements	Eigenvector centrality
	Katz status index
Subgraph–based measurements	Subgraph centrality
	Within–module *z*-score
Random–walk–based measurements	*k*-Step Markov
Social–capital–based measurements	Structural holes

The degree of a vertex *v* in a graph *G*=(*V*,*E*) is the number of connections it has. Here *V* is the set of vertices (genes or proteins) in the graph and *E* is the set of edges (links) in the graph. The distance *σ*_*vw*_ of a vertex *v* from another vertex *w* is the number of edges in the shortest path between them. A path in a graph is a sequence of edges that connect a sequence of vertices (no repeated vertices allowed). The walk is a path in which vertices or edges may be repeated.

The betweenness value of a vertex *v* is defined by the following equation:

$$B(v) = \sum\limits_{\substack{\scriptstyle{s} \in V \hfill\\ \scriptstyle{s} \ne v}} {\sum\limits_{\substack{\scriptstyle{t} \in V\\ \scriptstyle{t} \ne s,\\ \scriptstyle{t} \ne v}} {\frac{{\sigma_{st} (v)}}{{\sigma_{st} }}} }.$$

The numerator in the fraction shows the number of shortest paths joining *s* and *t* on which *v* is an intermediate vertex.

The closeness value of a vertex *v* is defined by the following equation:

$$C(v) ={\sum\limits_{\substack{\scriptstyle{t} \in V \\ \scriptstyle{t} \ne v}}{\frac{{1}}{{\sigma_{vt}}}}}.$$

The proximity prestige measure [12] could be measured as the ratio of the proportion of vertices that can reach *v* to the average path length of these vertices from *v*.

$$ P_{P} (v)= \frac{I_{v}/(|V|-1)}{{\sum\limits_{\substack{\scriptstyle{t} \in V\\ {\scriptstyle{t} \ne v }}} \sigma_{vt}/I_{v}}}, $$

where *I*_*v*_ is the number of vertices in the domain of node *v*.

Bary center score ranks each vertex of the graph depending on the total shortest path of the vertex. It computes the shortest path distances for each vertex in the graph and a score will be assigned for each vertex based on the lengths of the shortest paths that go through the vertex.

Clustering coefficient measures the degree of cohesiveness in a given graph. For a given vertex *v*, *C*_*cc*_(*v*) is defined as the ratio of actual number of edges *E*_*i*_ within its neighborhood and the maximum number of possible edges in that neighborhood.

The coreness value measures the set of vertices that are highly and mutually interconnected. The *k*-core is the largest subgraph, comprising vertices of a degree at least *k*, and is derived by recursively removing vertices with a degree lower than *k* until none remain.

Eigenvector centrality value expresses the centrality of a vertex as dependent on the centralities of its directly connected neighboring vertices. For a given undirected graph *G*=(*V*,*E*) and its adjacency matrix *A*, the eigenvector centrality is the eigenvector of the largest eigenvalue *λ*_*max*_ in absolute value. The eigenvector centrality *C*_*eiv*_ could be obtained from the following system of equations:

$$\lambda \overrightarrow{C_{eiv}} = A \overrightarrow{C_{eig}}. $$

Katz status index centrality ranks a vertex as highly important if a large number of vertices are connected to it. Both direct and indirect neighbors of a vertex contribute to its importance. Katz status index centrality (*C*_*ksi*_ is defined by the following equation:

$$\overrightarrow{C_{ksi}}=\left({\left(1- \alpha A^{T}\right)}^{-1}-I\right)\overrightarrow{1}, $$

*α* is a scaling factor. *A*^*T*^ is the transpose of *A*, *I* is an identity vector, $\overrightarrow {1}$ is a vector of ones.

Subgraph centrality value ranks vertices according to the number of times a given vertex participates in different connected subgraphs of a network [13]. For a vertex *v* in undirected graph *G*=(*V*,*E*) and its adjacency matrix *A*, the subgraph centrality for a node that has length of close walk *k* is computed as follows:

$$C_{sg}(v) ={\sum\limits_{k=0}^{\infty} \frac{(A^{k})_{vv}}{k!} }.$$

Within–module z-score measures how vertices are related. Modules could be organized in different ways. If *k*_*i*_ is the number of edges of vertex *i* to other vertices in its module *m*_*i*_, $\bar {k}_{m_{i}} $ is the average of *k* over all the vertices in *m*_*i*_, and $s_{m_{i}} $ is the standard deviation of *k* in *m*_*i*_, then, the within-module z-score is computed as follows:

$$z_{i}=\frac{k_{i}-\bar{k}_{m_{i}}}{s_{m_{i}}}. $$

The within–module z-score measures how well connected vertex *i* is to other vertices in the module.

*k*-Step Markov technique calculates the relative probability that the system will spend some time at any particular vertex, such that it is given the start set of roots *R* and ends after *k* steps. Let *P*_*u*,*v*_ be the probability of reaching *v* from *u* in one step. So, this probability is the weight of the edge between *u* and *v*. Then, let *N*(*u*) be the set of neighbor vertices of *u*. After that, the probabilities are constrained by the following equation.

$${\sum\limits_{\substack{u \in V\\ v \in N(u)}}{P_{u,v}}=1}.$$

Furthermore, a random walk is defined as a walk that starts at a particular vertex and traverses the graph based on *P*_*u*,*v*_. *k*-Step Markov centrality is the probability with which a random walk of length *k* brings a system to a particular vertex *v* [14], and could be obtained from the following equation.

$$ C_{ksm} (v,k)= P^{0} A^{k}, $$

where *P*^0^ is an initial probability distribution of the vertices in *G*, and *A* is the adjacency matrix of *G* containing the transition probabilities. In this study, we consider *k* to be 6.

To apply the structural hole concept, we identify nodes utilizing Burt’s aggregate constraint measure (Equation 2.7 in [15]). Burt’s structural hole argument is that social capital is created by a network in which individuals in the social network can broker connections between otherwise disconnected segments. This concept builds on a metaphor of ‘social capital’ that is made concrete with network models in which topological measures rank nodes by their connectivity and lack of redundancy. The argument further posits that since there is some cost of maintaining connections, non-redundancy increases the influence of a node.

### Breast cancer signature genes

In this study, three major databases have been utilized to identify the breast cancer signature genes (genes that influence breast cancer disease):

- The Genetic Association Database (GAD) [16].
- The Mammalian Phenotype (MP) [17].
- The Human Phenotype Ontology [18].

We have extracted 451 genes that related to breast cancer from the databases mentioned above. We fed this gene data as class labels into classifiers. Thereby the class labels in the dataset are represented as ‘Yes’ (genes that influence breast cancer disease) and ‘No’ (genes that do not influence breast cancer disease).

### Synthetic minority oversampling technique

Synthetic Minority Oversampling Technique (SMOTE) [19] is a sampling approach used to transform an imbalanced dataset to a balanced one. A dataset can be considered imbalanced if there is one group of observations with a very minimum number of samples compared to the other group of observations in the same dataset. It is well known that a machine learning classifier cannot perform well if the dataset is highly imbalanced. The dataset we used in this study is imbalanced by nature and hence application of SMOTE could transform the dataset to a balanced one.

**Fig. 1:**
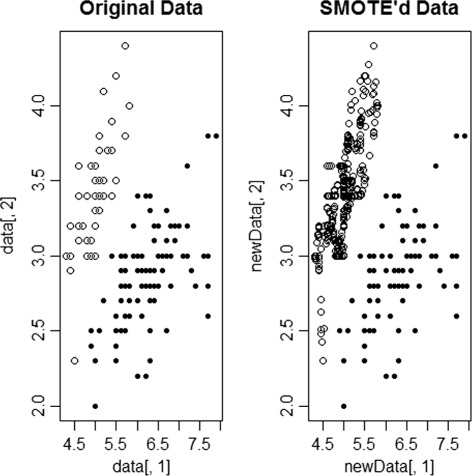
SMOTE’d data example (sample data)

The SMOTE approach over–samples the minority class by creating synthetic samples rather than over–sampling with replacement. In other words, the positive (minority) samples are over–sampled with replacement to match the number of negative (majority) samples, as shown in Fig. 1. This method operates in ‘feature space’ rather than ‘data space’: i.e each feature is over–sampled. In line with this, the minority class is over-sampled by taking each sample belonging to the minority class and generating synthetic samples to increase the sample size. This is done using a *k*-nearest neighbor algorithm among the minority samples. The sample that appears to be the closest *k* neighbor is joined together to generate a new sample.

### Classification models

In this study we have used two different classifiers: Decision Tree Bagger (DTB) [20] and Random under sampling boost RUSBoost [21] in order to classify the data, based on the extracted topological measures as features and breast cancer signature genes as the class label.

Decision Tree Bagger employs a classic decision tree as the classifier and then a bagging methodology is used to further enhance the classification performance of the classifier. A decision tree is a widely used classifier that divides the dataset such that the impurity level in each partitioned dataset is reduced when compared to the dataset that has been partitioned. The impurity level of a dataset is measured using the class label of each of the records. The most popular measurement for measuring impurity level is the Gini Index. Following a tree structure view, the source dataset is considered as a root node of the tree, while each partitioned dataset is considered as a child node that is rooted at the corresponding root node. Dataset partition is repeated at each of the sub-partitions with the aim of achieving a pure partitioned data at the leaf node of each of the branches of the tree. Once the tree is induced from the training dataset, traversing the tree from the root to each of the leaf nodes generates rules. These rules are then applied to classify an unknown dataset. Since the decision tree is induced from the training dataset, the tree structure might vary with varying sets of data of the same problem. Hence, the performance of the respective decision tree also could vary. To overcome this and achieve an enhanced classification performance a number of bootstrap replicas of the dataset are generated. This process of generating multiple replicas of the dataset, by varying sets of data in each of the datasets, is called the bagging or bootstrap methodology. Through application of the bagging methodology, the resultant individual replica of the training dataset is used to induce a decision tree. Thus, there will be as many decision trees as there are generated dataset replicas. The bagging replica could be sampled randomly choosing from *N* observations out of *N* with replacement, where *N* is the total data events in the dataset. Furthermore, the average of the classification performances from individual trees is considered as the output of the decision tree bagger.

Random Under Sampling Boost (RUSBoost) decision tree is another approach used to enhance the performance of the base decision tree classifier to better deal with an imbalanced dataset. In this approach, the data that belongs to the minority class is considered as the basic population, while data belonging to the majority class is under–sampled, such that the data for each of the classes becomes balanced. Let us consider that there are observations that belong to the minority class in the training data. Following the RUSBoost approach, these *N* observations are considered as the basic population for sampling. Thus, a total *N* observation from the data belonging to the majority class is sampled. Note: if there is more than one class that is considered as a majority class, *N* observations are sampled from each of the classes. All of the sampled data is merged with the *N* observations from the minority class to form a balanced dataset. After achieving a balanced dataset, a decision tree is induced using this dataset.

### Performance metrics

We consider several measures in order to evaluate each classifier performance:

Accuracy (ACC) is one of the most widely used performance metrics in evaluating a classifier. ACC is defined by the following equation:

$$\text{ACC} = \frac{\left(\text{TP}+\text{TN}\right)}{\mathrm{N}}, $$

where (TP+TN) represents all samples that are classified correctly (both True Positive (TP) and True Negative (TN)) and *N* is the total number of samples available (N = (TP + TN) + (FP + FN)). (FP+FN) represents all samples that are classified incorrectly (both False Positive (FP) and False Negative (FN)).

Positive predictive value (PPV) is the proportion of cancerous genes in the prediction that are correctly predicted as cancerous genes:

$$\text{PPV} = \frac{\text{TP}}{\left(\text{TP}+\text{FP}\right)}. $$

Sensitivity (SN) refers to the proportion of cancerous genes which are correctly predicted as cancerous and the total cancerous genes:

$$\text{SN} = \frac{\text{TP}}{\left(\text{TP}+\text{FN}\right)}. $$

Specificity (SP) refers to the proportion of non-cancerous genes that are correctly eliminated and the total non-cancerous genes:

$$\text{SP} = \frac{\text{TN}}{\left(\text{TN}+\text{FP}\right)}. $$

F-measure (F) is the harmonic mean of sensitivity and positive predictive value, which is defined as:

$$\mathrm{F} = \frac{\left(2 \cdot \text{SN} \cdot \text{PPV}\right)}{\left(\text{SN}+\text{PPV}\right)}. $$

Geometric mean (Gm) has been introduced to overcome the problem that is associated with the accuracy metric in imbalanced dataset learning:

$$\text{Gm} = \sqrt{(\text{SN} \cdot \text{SP})}. $$

The receiver operating characteristic (ROC) curve [22] is a well known performance measurement metric used to evaluate the performance of a classifier, particularly when the dataset is highly imbalanced. The ROC curve can be generated by considering a two-dimensional Cartesian plot, where the x-axis represents the amount (1-SP) and the y-axis represents SN. It should be noted that by varying the threshold level of classifying the data into two classes (e.g. either 1 or 0), the above mentioned measures will also vary. Hence the ROC plot reflects these variations in terms of both Sensitivity and Specificity. In summary, through analysis of the ROC plot it can be easily identified which threshold level provides the best performance for a classifier. It is worth mentioning here that the best possible performance for a classifier can be achieved if both Sensitivity and Specificity yield 100 %. In other words, the ROC curve that exactly matches the upper part of the ROC space yields the best performance. Hence, the closer the curve to the upper part of the ROC space, the better the performance is. Alternatively, the area under the curve can reveal the quality of the classifier’s performance. If the curve covers the whole ROC space, the classifier is called the perfect classifier. As such, the area under the curve (AUC) can also be used as an indication of classifier performance. An AUC value equal to 1 is called the best classifier, while anything close to 1 can be considered as good as that of the perfect classifier. An AUC value less than 0.5 is considered to be a random classifier performance.

### Validation

To achieve a generalized performance of the proposed method, we applied the well known *k*-fold cross validation schema. In this schema, the dataset is divided into *k* equal partitions and a computational model is generated using *k*−1 partitioned datasets, while the *k*^*th*^ partitioned dataset is kept untouched in order to test the model later. These steps are repeated *k* times such that each individual data is used to test the efficacy of the proposed model. It is worth mentioning that for k-fold partition, a total k number of models with varying training datasets are generated. As our proposed model consists of identification of features that are based on the performance of the proposed model, while selecting features we considered only the total *k*−1 partitions of dataset by keeping the data belonging to the *k*^*th*^ partition aside. By doing so we achieve a more general performance of the proposed model without having any bias towards any class of data.

## Results and discussion

In this study three public networks are utilized to extract network topological features: a) the gene co-expression network obtained from Hedenfalk et al. [23]; b) the protein interaction network of *Homo Sapiens* obtained from the BioGrid database (version 3.4.132) [24]; and c) the integrated functional interaction network which made publicly available by Wu et al. [11]. We compare the performance of the classification models in predicting the phenotype-gene association using features extracted from these networks. We report the performance measures that were mentioned earlier. Table 2 presents a comparison of the performance of classification models.

**Table 2:** Comparison of classification results which adapt SMOTE sampling

Classifier	# Metric	Public networks		
		GCN	PPI	FI
DTB	ACC	.89±.02	.88±.02	.90±0.02
	F	.89±.02	.89±.02	.90±0.02
	AUC	.89±.02	.88±.02	.90±0.02
	G-Means	.89±.02	.88±.02	.90±0.02
RUSBOOST	ACC	.80±.04	.82±.02	.82±0.03
	F	.80±.04	.83±.02	.82±0.02
	AUC	.80±.04	.82±.02	.82±0.03
	G-Means	.80±.04	.82±.02	.81±0.03

We applied 10−fold cross validation schema. We then compute the 95 *%* confidence interval for the mean with the following formula: *Q*=*M*±*Z*_.95_*σ*_*M*_, where *Z*_.95_ is the number of standard deviations extending from the mean of a normal distribution required to contain 0.95 of the area and *σ*_*M*_ is the standard error of the mean. Clearly, the DTB classification model, which adapts SMOTE sampling and uses topological features extracted from the integrated functional interaction network (FI), has the highest *G*-Mean value (0.90±0.02), as illustrated in Table 2. A high *G*-Mean value indicates that a high proportion of the cancerous genes (the breast cancer genes’ signature) are predicted correctly. On the other hand, the DTB classification models that adapt SMOTE sampling and use topological features extracted from the other two networks — GCN and PPI — have lower *G*-Mean values of (0.89±0.02) and (0.88±0.02), respectively. This indicates that using an integrated functional interaction network can reveal more information about phenotype-gene association in breast cancer. RUSBoost has similar results but has one major drawback: the RUSBoost uses its own sampling method, which creates a conflict with the SMOTE sampling method.

**Fig. 2:**
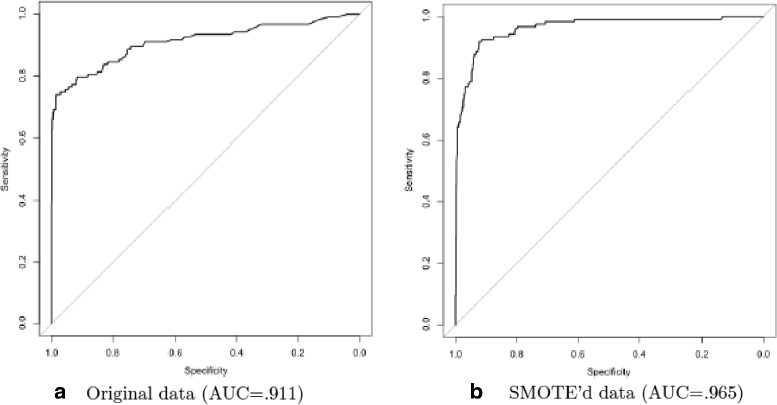
Comparison of classification results on the FI network data

Moreover, we compare the performance of the DTB classification model that adapts SMOTE sampling with the one that does not adapt SMOTE sampling. The performances were computed using areas under the ROC curves (AUC). Clearly, the DTB classification that adapted SMOTE sampling has the largest area under the ROC curve (AUC = 0.965), as shown in Fig. 2.

In addition, we compute the significant level of each of the selected topological measurements by using two well known statistical measurements: accuracy and the Gini Index. Clearly, the Gini Index outweighs the accuracy score (as illustrated in Table 3). To overcome this problem we compute the geometric mean of accuracy and Gini Index as a combined score. We compute the accuracy, the Gini Index and the combined score based on the DTB classification model that adapts SMOTE sampling and using topological features extracted from the integrated functional interaction network. The results are illustrated in Table 3. It can be seen from that table that ‘Structural Holes’ and ‘Degree’ features outperform the other features by a significant margin in terms of combined score values. In addition, a backward elimination method has been computed that identifies a subset of five features as important features in predicting phenotype-gene association. The identified features are ‘Structural Holes’, ‘Degree’, ‘Coreness’, ‘*k*-Step Markov’ and ‘Subgraph’.

**Table 3:** Feature importance analysis: Accuracy, Gini Index, and the combined score are listed

Topological measures	Accuracy	Gini index	Combined score
Structural holes	0.3081	579.9545	13.37
Degree	0.3088	578.1108	13.36
Coreness	0.3056	474.7823	12.05
*k*-Step Markov	0.2958	371.1454	10.47
Subgraph centrality	0.3032	354.3712	10.36
Within–module *z*-score	0.2704	291.5019	8.88
Katz status index	0.2882	259.1472	8.64
Closeness	0.2943	227.2495	8.18
Proximity prestige	0.2962	222.5109	8.12
Eigenvector centrality	0.2834	230.7507	8.09
Betweenness	0.2731	230.3441	7.93
Bary center score	0.2742	118.4802	5.70
Clustering coefficient	0.0632	0.3585	0.15

Finally, we investigate genes that not classified correctly, particularly the ones from the group where genes are not cancerous but the method misclassifies them as cancerous genes. Table 4 illustrates some of these genes. Each gene is listed according to its symbol, name and related OMIM disease. The table shows that our method is capable of identifying new genes that may be related to breast cancer.

**Table 4:** List of some genes that are misclassified by the method as breast cancer related genes

Gene symbol	Gene name	OMIM disease
*CD4*	CD4 molecule	CD4+ lymphocyte deficiency
*APP*	amyloid beta (A4) precursor protein	Alzheimer disease 1, Amyloidosis, Dementia, early-onset progressive, autosomal recessive,
*CDK2*	cyclin-dependent kinase 2	A novel susceptibility locus for type 1 diabetes.
*FN1*	fibronectin 1	Glomerulopathy with fibronectin deposits.
*IRF1*	interferon regulatory factor 1	Gastric cancer, Macrocytic anemia, Myelodysplastic syndrome, preleukemic, Myelogenous leukemia, acute, Nonsmall cell lung cancer.
*PSEN1*	presenilin 1	Alzheimer disease, Cardiomyopathy, Pick disease.
*STAT1*	signal transducer and activator of transcription 1	Mycobacterial infection, atypical, familial disseminated.
*SLC25A3*	solute carrier family 25	Micochondrial phosphate carrier deficiency.
*SOS1*	son of sevenless homolog 1	Fibromatosis, gingival, Noonan syndrome 4.

## Conclusions

We have compared various topological measures that have the potential to identify phenotype-gene association for breast cancer. We have extracted thirteen features from publicly available gene co-expression networks and protein interaction networks. We have used two classification models to investigate the phenotype-gene association in breast cancer. Moreover, we have applied this approach to the integrated functional network of PPI and gene expression in order to investigate the hidden pattern of breast cancer that might not be revealed in the protein networks or gene co-expression networks.

In conclusion, our approach is capable of effectively detecting the phenotype-gene association in breast cancer.
